# Deregulated Cardiac Specific MicroRNAs in Postnatal Heart Growth

**DOI:** 10.1155/2016/6241763

**Published:** 2016-12-25

**Authors:** Pujiao Yu, Hongbao Wang, Yuan Xie, Jinzhe Zhou, Jianhua Yao, Lin Che

**Affiliations:** ^1^Department of Cardiology, Tongji Hospital, Tongji University School of Medicine, Shanghai 200065, China; ^2^Department of Cardiology, Yangpu Hospital, Tongji University School of Medicine, Shanghai 200090, China

## Abstract

The heart is recognized as an organ that is terminally differentiated by adulthood. However, during the process of human development, the heart is the first organ with function in the embryo and grows rapidly during the postnatal period. MicroRNAs (miRNAs, miRs), as regulators of gene expression, play important roles during the development of multiple systems. However, the role of miRNAs in postnatal heart growth is still unclear. In this study, by using qRT-PCR, we compared the expression of seven cardiac- or muscle-specific miRNAs that may be related to heart development in heart tissue from mice at postnatal days 0, 3, 8, and 14. Four miRNAs—miR-1a-3p, miR-133b-3p, miR-208b-3p, and miR-206-3p—were significantly decreased while miR-208a-3p was upregulated during the postnatal heart growth period. Based on these results, GeneSpring GX was used to predict potential downstream targets by performing a 3-way comparison of predictions from the miRWalk, PITA, and microRNAorg databases. Gene Ontology (GO) and Kyoto Encyclopedia of Genes and Genomes (KEGG) analysis were used to identify potential functional annotations and signaling pathways related to postnatal heart growth. This study describes expression changes of cardiac- and muscle-specific miRNAs during postnatal heart growth and may provide new therapeutic targets for cardiovascular diseases.

## 1. Introduction

Although the heart is recognized as a nearly terminally differentiated organ, its weight increases rapidly by approximately 20-fold from birth to adulthood (i.e., the period of postnatal heart growth). The development of the heart is a precise and complex process that is subjected to the regulation of many molecules.

MicroRNAs (miRNAs, miRs) are endogenous, highly conserved, short noncoding RNAs that can posttranscriptionally regulate gene expression by binding to complementary sequences on target mRNAs [1–5]. miRNAs play important roles in many biological processes, including development, differentiation, proliferation, apoptosis, metabolism, and tissue remodeling [6], and the expression patterns of miRNAs are restricted spatially and temporally in different tissues and during various developmental stages. The high conservation of all types of miRNAs is closely related to their important functions, especially regarding the evolution of their target genes.

Overwhelming studies have demonstrated that miRNAs exert functions during development and that regulating the expression of their target genes is essential for the development of multiple systems, including the gastrointestinal system and neural system [7–12]. However, the role of miRNAs in postnatal heart growth is seldom discussed. The integration and interaction of the effects of miRNAs on the heart provide a regulatory guarantee of cardiac gene expression [13]. These miRNAs may also have a potential role in regulating postnatal heart growth.

In the present study, we explored the temporal pattern of cardiac- and muscle-specific miRNAs during postnatal heart growth as well as the potential target genes of these miRNAs as regulators of postnatal heart growth. The data will provide novel insights for the development of postnatal heart growth.

## 2. Materials and Methods

### 2.1. Mice

Male and female wild-type C57BL/6 mice were purchased from the Shanghai Laboratory Animal Center (SLAC, Shanghai, China) and housed in specific pathogen-free (SPF) conditions on a 12 h light/12 h dark cycle in a temperature-controlled room (21–23°C). All animal experiments were conducted under the guidelines of the humane use and care of laboratory animals for biomedical research published by the National Institutes of Health (no. 85-23, revised 1996). This study was approved by the Local Ethic Committee of Animal Experiments at Tongji University.

### 2.2. Quantitative Reverse Transcriptase-Polymerase Chain Reaction (qRT-PCR)

To collect heart tissues for qRT-PCR analysis, mice were sacrificed by cervical dislocation at postnatal days 0, 3, 8, and 14. The tissues were isolated, rapidly removed, frozen fresh in liquid nitrogen, and stored at −80°C until use. Total RNA was extracted using TRIzol reagent (Invitrogen). For miRNA analysis, cDNA was generated, and the amplification and detection of specific products were performed on an ABI 7900 qPCR System. U6 was used as an internal control to normalize miRNA expression. Primers sequences of miRNAs (forward, 5′-3′) were designed as follows: miR-1a-3p, ACGATGGAATGTAAAGAAGT; miR-133a-3p, ACGATTTGGTCCCCTTCAAC; miR-133b-3p, ACGATTTGGTCCCCTTCAAC; miR-208a-3p, ACGAATAAGACGAGCAAAAA; miR-208b-3p, ACGAATAAGACGAACAAAAG; miR-206-3p, ACGATGGAATGTAAGGAAGT; miR-499-5p, ACGATTAAGACTTGCAGTG; common reverse, GTGCAGGGTCCGAGGT, primers sequences of U6 (forward and reverse, 5′-3′), GCTTCGGCAGCACATATACTAAAAT, and CGCTTCACGAATTTGCGTGTCAT. Expression values were presented as fold change 2^−Δ(ΔC_T_)^ where ΔC_T_ = (C_T_ gene of interest −C_T_ internal control).

### 2.3. Bioinformatic Analysis

GeneSpring GX software was used to predict the target genes of the miRNAs, and these predictions were compared with 3 databases (microRNAorg, PITA, and miRWalk) and integrated into a Venn diagram to demonstrate interactions among the databases. The Gene Ontology (GO) database was used to describe 3 attributes of the identified gene products: molecular function, subcellular location, and related biological processes. Molecule and gene networks were analyzed by the Kyoto Encyclopedia of Genes and Genomes (KEGG) pathway database.

### 2.4. Statistical Analysis

One-way ANOVA was conducted with a Bonferroni's post hoc test. Data analyses were performed using SPSS 20.0 software, and all statistical tests were two-sided. *p* < 0.05 was considered to be statistically significant.

## 3. Results

### 3.1. Distinct Temporal miRNA Expression Profiles in Hearts during Postnatal Cardiac Development in Mice

**Figure 1:**
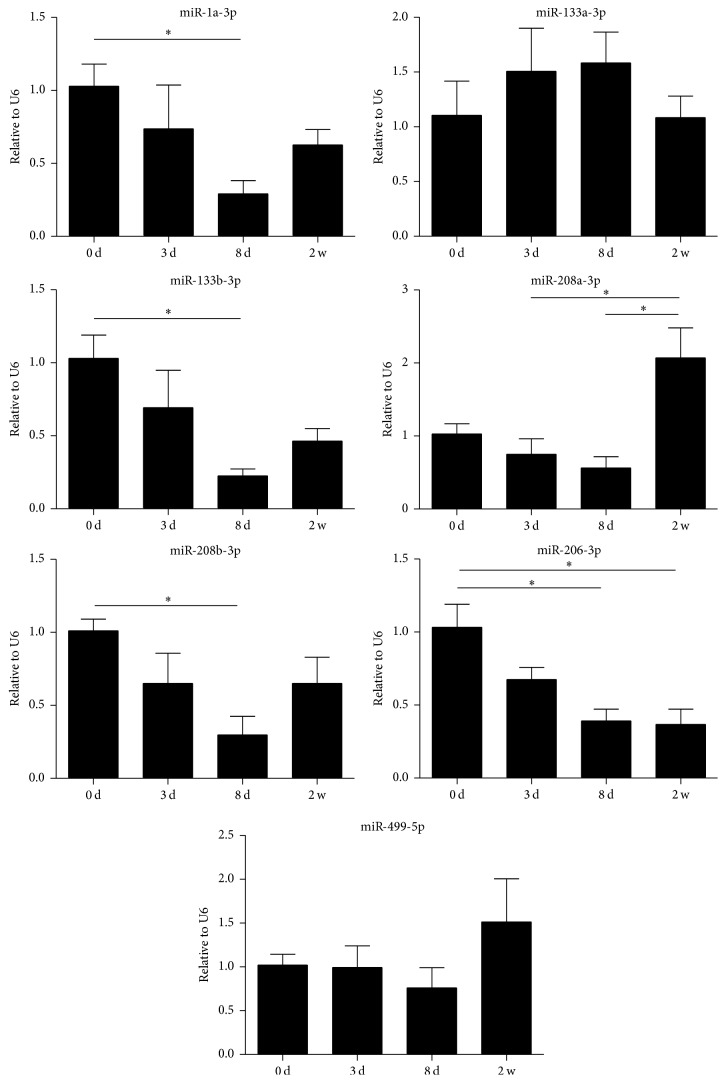
Validation of time-varying miRNAs using qRT-PCR. miRNAs levels in mice heart at days 0, 3, 8, and 14 are shown. *∗* indicates significant differences (*p* < 0.05) between two groups.

The period of cardiac muscle postnatal growth until 14 days after birth is defined as the maturation and differentiation stage. To test whether the observed cardiac or muscle miRNA expression profiles changes are temporal, we compared the miRNA expression profiles of mice hearts at postnatal days 0, 3, 8, and 14 by using qRT-PCR and found miR-1a-3p, miR-133b-3p, miR-208b-3p, and miR-206-3p were significantly decreased while miR-208a-3p was upregulated (Figure 1).

### 3.2. Target Gene, GO, and Pathway Analysis

**Figure 2:**
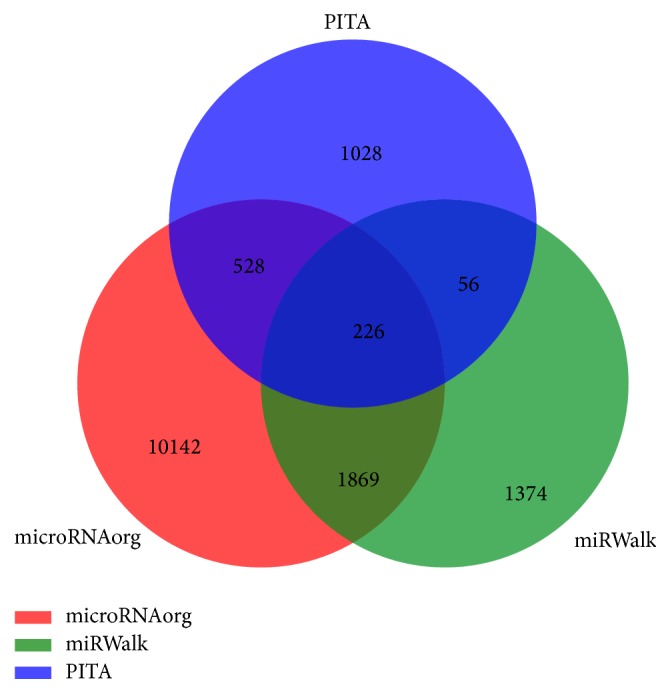
A 3-way comparison of predictions from microRNAorg, miRWalk, and PITA. The red, green, and blue sets stand for target genes predicted by databases microRNAorg, miRWalk, and PITA, respectively.

We used the microRNAorg, PITA, and miRWalk databases to predict the target genes of differentially expressed miRNAs in heart tissues at different time points during postnatal development by using GeneSpring software. Next, we examined the overlap of the resulting gene lists among these 3 databases and constructed a Venn diagram. There were 226 overlapping genes that were most likely to be targets of miRNAs during heart postnatal growth listed in the analysis results (Figure 2).

**Figure 3:**
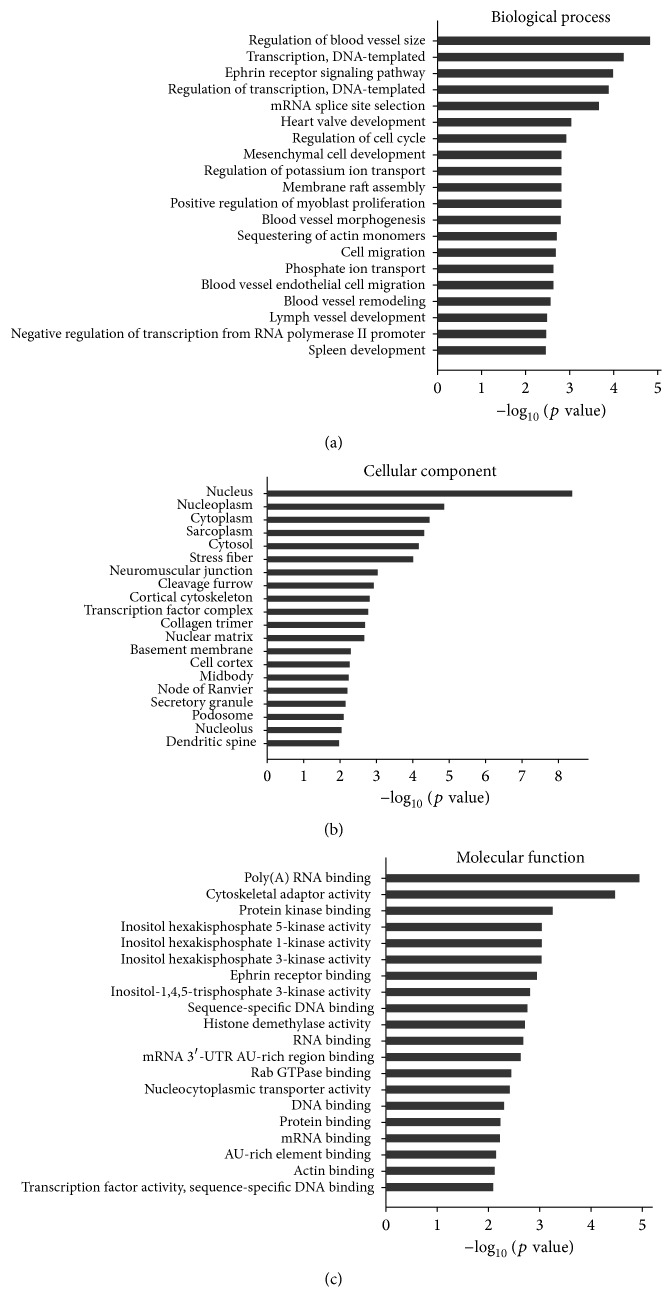
GO analysis for differentially expressed mRNAs. (a–c) GO analysis according to biological process, cellular component, and molecular function, respectively, ranked by enrichment score (−log_10_⁡ (*p* value)).

The function of up- and downregulated genes was classified by Gene Ontology (http://geneontology.org/) based on 3 attributes: molecular function, biological processes, and subcellular location. In this study, differentially expressed mRNAs were enriched in numerous biological processes including the regulation of blood vessel size, cell cycle, and migration; transcription; DNA replication; ephrin receptor signaling; heart valve development; and positive regulation of myoblast proliferation (Figure 3(a)). Similarly, the nucleus, nucleoplasm, and cytoplasm were the identified cellular components affected (Figure 3(b)), whereas the affected molecular functions include the following: poly(A) RNA binding, cytoskeletal adaptor activity, and protein kinase binding (Figure 3(c)).

**Figure 4:**
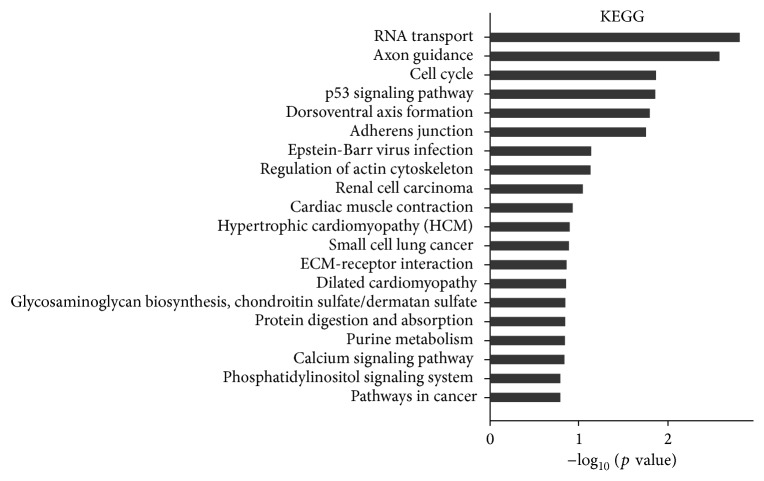
Pathway analysis based on the KEGG database. Ranked by enrichment score (−log_10_⁡ (*p* value)).

Moreover, KEGG pathway analysis identified the following significantly (*p* < 0.05) affected pathways: cAMP signaling, vascular smooth muscle contraction, regulation of actin cytoskeleton, neurotrophin signaling, and cGMP-PKG signaling (Figure 4).

## 4. Discussion

The heart is the first functional organ during embryonic development and plays an important role in the growth and maintenance of higher organisms. In contrast to most organs, the heart is more sensitive to small changes in the gene expression during development. Even subtle biological perturbations in cardiac structure may result in catastrophic consequences [14]. Elucidating the effects of those subtle perturbations is key to fully understanding the molecular mechanisms of heart development.

MiRNAs, which are involved in a variety of mechanisms that regulate gene expression, play vital roles in heart formation and cardiovascular disease [15–18]. Increasing evidence has indicated the diagnostic value and clinical implications of several miRNAs in heart diseases, such as miR-433, miR-21, miR-378, and miR-940 [19–21]. An increasing number of miRNAs with different functions in heart development have also been identified, including miR-1, miR-208, miR-133, miR-206, miR-126, miR-143, miR-145, and miR-499; from this group, we analyzed the 7 miRNAs most relevant to postnatal heart growth. Here, we found that the time after birth is responsible for the changes of the observed miRNA expression profiles. These results indicated that the miRNA expression levels present significant differences as a restricted temporal expression pattern during different developmental stages.

Previous studies showed that miR-1 and miR-133 are highly correlated with heart development, and miR-1 was the first miRNA to be implicated in heart development [22]. Several studies showed that these two gene clusters were related to processes involved in cardiac muscle development, including mediated embryonic development, embryonic stem cell differentiation, proliferation and apoptosis, sarcomere disarray, cardiac fibrosis, cardiac rhythm control, and remodeling. [23–26]. However, more subtle regulation of these gene clusters results in antagonistic effects in contrast to their more established roles [27, 28]. The functions of the identified up- and downregulated genes were classified by GO analysis. The results showed a series of potential regulatory functions in cell development, cytoskeleton formation, and angiogenesis, which suggest that these miRNAs may be involved in postnatal heart development. When excluding the two miRNAs with the strongest influence on gene expression in the heart, there are many other miRNAs that have been functionally analyzed in the cardiovascular system and commonly promote more balanced development of the heart. Finally, only fully understanding and appreciating the microRNA regulatory networks in cardiovascular development can provide a new perspective on heart disorders as well as new therapeutic targets for congenital heart disease.

In conclusion, the present study explores the temporal pattern of several cardiac- and muscle-specific miRNAs in postnatal heart development. By improving our understanding of cardiac growth, these results provide new insights in the diagnosis and treatment of heart diseases.
